# A case of successful pregnancy in a septate uterus after discharge of decidual tissue in the second trimester

**DOI:** 10.1002/ccr3.4042

**Published:** 2021-03-11

**Authors:** Mari Uomoto, Soichiro Obata, Ami Yumoto, Sayuri Nakanishi, Yukiko Sasahara, Masako Otani, Etsuko Miyagi, Shigeru Aoki

**Affiliations:** ^1^ Perinatal Center for Maternity and Neonates Yokohama City University Medical Center Yokohama Japan; ^2^ Department of Pathology Yokohama City University Medical Center Yokohama Japan; ^3^ Department of Obstetrics and Gynecology Yokohama City University Hospital Yokohama Japan

**Keywords:** bleeding, decidua, pregnancy, uterine anomalies

## Abstract

In pregnant patients with a divided uterine cavity, the decidual tissue on the nonpregnant side may be discharged prior to the delivery of the fetus. The pregnancy can continue if the uterine contractions and vaginal bleeding are controlled and the fetus is not in distress.

## Graphical abstract

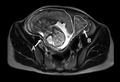

## 1 INTRODUCTION

Uterine malformation results from abnormal fusion of the left and right Mullerian ducts in early development,^1^ and it occurs in 5.5% of the general population.^2^ Uterine malformation can be the cause of miscarriage, preterm delivery, and vaginal bleeding during pregnancy.^3^, ^4^ Here, we report a case of a successful pregnancy in a septate uterus wherein tissue with decidual changes was discharged from the nonpregnant side of the uterus with a significant amount of bleeding during the second trimester. Vaginal bleeding during pregnancy is common in malformed uteruses with nonpregnant cavities; however, there are few reports of continuing pregnancy after discharge of tissue with decidual change. The patient provided informed consent for the publication of this case report.

## 2 CASE PRESENTATION

**FIGURE 1:**
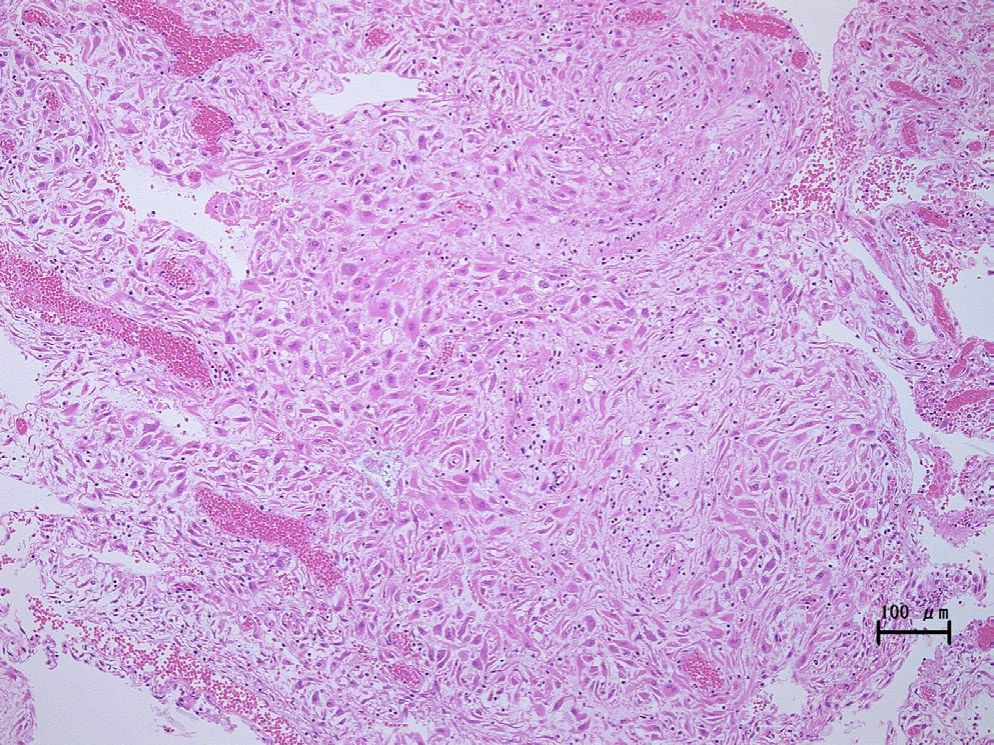
Pathology of discharged tissue. The discharged tissue has decidual changes and no components of amniotic or villous membranes (hematoxylin and eosin stain, 100× magnification; scale bar is 100 μm)

**FIGURE 2:**
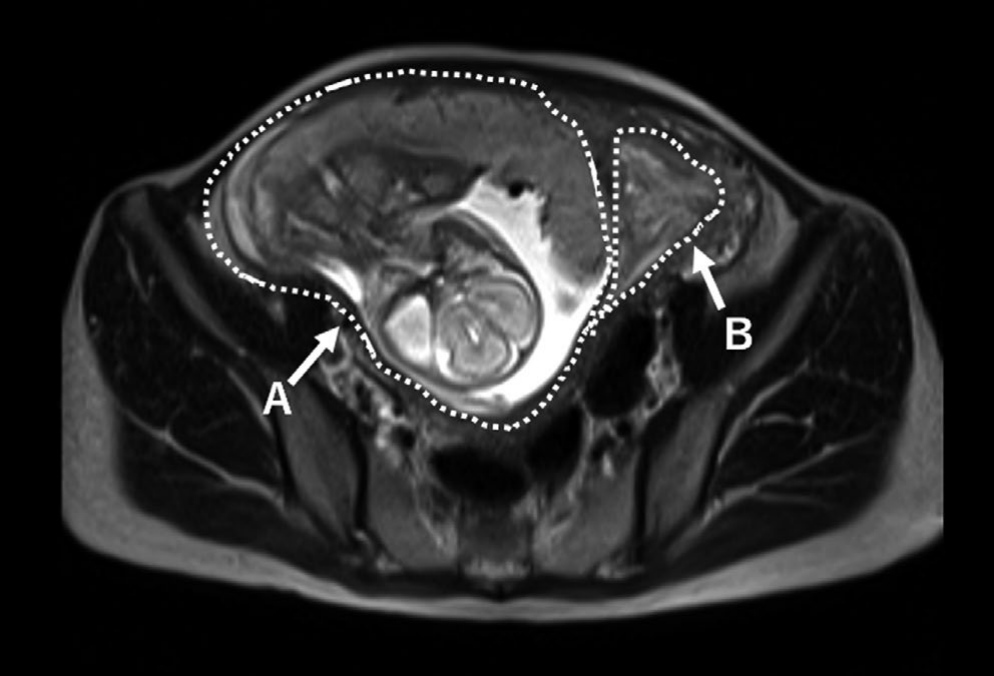
Magnetic resonance imaging (MRI) of septate uterus at 21 wk and 5 d of gestation. A, A T2‐weighted MRI reveals a fetus in the right side of the uterus with no signs of hematoma or thickened placenta. B, The left side of the uterus contains a hematoma

A 31‐year‐old pregnant woman with a history of uterine malformation presented with vaginal bleeding and uterine contractions. She had a history of a resected vaginal septum at age 12. The ESHRE/ESGE (the European Society of Human Reproduction and Embryology/the European Society for Gynaecological Endoscopy) classification of the uterine malformation was U2C1V2 (Figure S1).^5^ Similarly, the patient had a history of successful pregnancy delivered vaginally at 38 weeks and 3 days of gestation. The current pregnancy was conceived spontaneously, and the patient's due date was determined using her last menstrual period by another hospital. At 21 weeks of gestation, the patient experienced vaginal bleeding and uterine contractions. Approximately 200 g of blood was discharged from the cervical os, and regular uterine contractions with pain were observed every 10 minutes. The patient was admitted to the hospital, and tocolysis was started. At 21 weeks and 3 days of gestation, a specimen of placenta‐like tissue with a diameter of 5 cm was discharged from the uterus with a significant amount of blood. A pathological examination revealed that the tissue had decidual changes (Figure 1). The bleeding gradually decreased, the uterine contractions were controlled, and we could confirm the fetal well‐being with ultrasound sonography. Magnetic resonance imaging showed the fetus in the right uterine cavity and a hematoma in the left uterine cavity (Figure 2). The patient was subsequently referred to our hospital at 22 weeks of gestation with significantly decreased bleeding and contractions every 10‐15 minutes. A transvaginal ultrasound revealed a cervical length of 30 mm, a hematoma in the nonpregnant uterine cavity, and no signs of placental abruption in the pregnant uterine cavity. No signs of fetal growth restriction or oligohydramnios were observed. She did not have any complications or family history related to coagulopathy. The hemoglobin and hematocrit values were 10.4 g/dL and 31.2%, respectively, and blood transfusion was not needed. The amount of bleeding and frequency of uterine contractions continued to decrease throughout the hospital stay.

Tocolysis was discontinued at 23 weeks of gestation, and the patient was discharged at 24 weeks of gestation. She had regular outpatient follow‐up every 2 weeks. At 30 weeks and 5 days of gestation, the patient presented with vaginal bleeding and was observed to have a shortened cervical length of 16 mm. She was admitted to the hospital and administered betamethasone.

At 33 weeks and 6 days of gestation, the rupture of membrane occurred spontaneously, and spontaneous labor started subsequently. A male infant weighing 1,684 g (appropriate for gestational age) was delivered. The infant's Apgar scores were 9 and 9 at 1 and 5 minutes, respectively. The postpartum period was uneventful, and the patient was discharged on postpartum day 4. Although the neonate was admitted to the neonatal intensive high care unit (NICU), he developed without any complications and was discharged home at the age of 66 days.

## 3 DISCUSSION

When pregnancy occurs in a uterus with a divided cavity, decidual tissue can be discharged with vaginal bleeding from the nonpregnant uterine cavity prior to the fetus delivery. When uterine contractions and bleeding are controlled properly and the fetal well‐being is confirmed, the pregnancy can continue even in cases of severe blood loss.

In a normal uterus, the endometrium develops into decidua and becomes thicker due to the presence of progesterone during the first trimester of pregnancy. In the second and third trimesters, the decidua is compressed against the uterine wall due to the enlargement of the fetal and villous tissues. As gestation progresses, the decidua thins and fuses with the amniotic and villous membranes to form a thin fetal membrane.^1^ In this patient, a pathological examination of the discharged placenta‐like tissue revealed decidual changes without components of amniotic or villous membranes (Figure 1). In patients with uterus malformations wherein the uterine cavity is divided into pregnant and nonpregnant sides, the tissue with decidual change on the nonpregnant side is not compressed by the amniotic cavity and remains thick. This tissue can be spontaneously discharged. In a previous report, we presented a patient with spontaneous discharge of the decidual tissue during the second trimester that resulted in the termination of pregnancy due to an excessive amount of bleeding.^6^ Though these cases are rare, discharge of tissue with decidual changes in a separate uterine cavity should be considered when placenta‐like tissue is discharged with a large amount of bleeding during pregnancy.

Vaginal bleeding and uterine contractions in the second trimester may be due to various conditions, including placental previa, placental abruption, chronic abruption‐oligohydramnios sequences, and subchorionic hematoma.^7^, ^8^, ^9^, ^10^ Among these conditions, placental abruption has a high mortality rate^11^ and should be excluded. In this patient, vaginal bleeding and uterine contractions were well controlled, the fetal well‐being was confirmed with ultrasound sonography, there were no signs of hematoma or thickened placenta on the pregnant side of the uterus, and blood tests revealed no coagulation dysfunction.^7^ Therefore, the possibility of placental abruption was considered low, and the pregnancy was continued, resulting in a healthy male infant. Although a significant amount of bleeding may occur when the decidual tissue is discharged, expectant management can ensure a healthy pregnancy. If discharge of tissue with decidual changes occurs at a more advanced gestational age, fetal heart monitoring or the fetal well‐being would have to be followed up more carefully with ultrasound sonography.

## CONFLICT OF INTEREST

I and my co‐authors have no conflict of interest.

## AUTHOR CONTRIBUTIONS

MU: Contributed to the treatment and first draft and finalization of the manuscript. SO: Contributed to the first draft and finalization of the manuscript. AY and SN: Contributed to patient care. YS and MO: Contributed to pathological examination and manuscript regarding the pathological image. EM and SA: Supervised the case report.

## ETHICAL APPROVAL

Prior to submission, appropriate consent for publication of images and data has been obtained.

## Supporting information

Fig S1

## Data Availability

Data sharing not applicable to this article as no datasets were generated.
