# Supplementary figures and images for: A case of successful pregnancy in a septate uterus after discharge of decidual tissue in the second trimester
Source: Clin Case Rep. 2021 Mar 11;9(4):2382–4. doi: 10.1002/ccr3.4042 (PMC8077306; doi:10.1002/ccr3.4042)

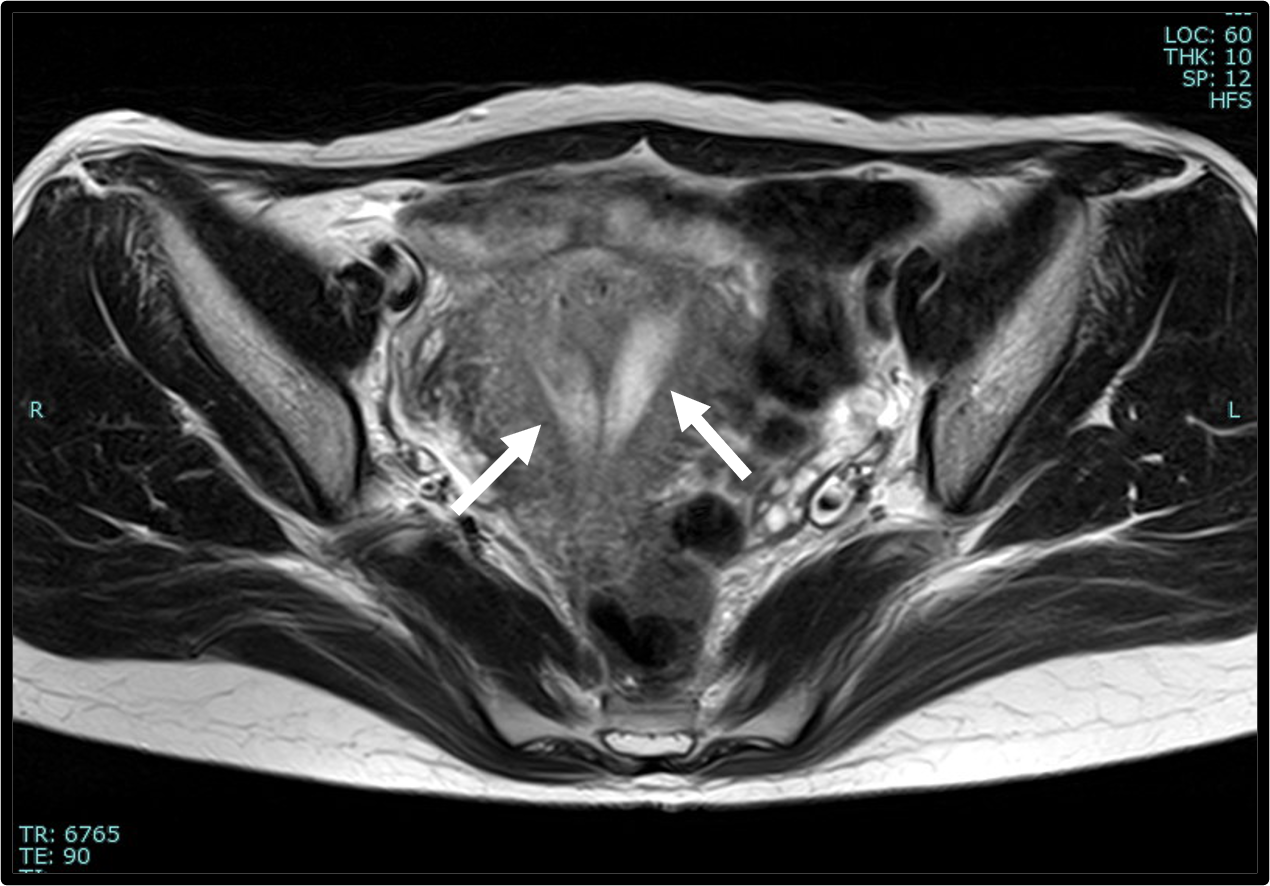

Supplement: Supplementary file 1 — Fig S1 [file CCR3-9-2382-s001.png]
